# Evaluation of Pharmacokinetics, and Bioavailability of Higher Doses of Tocotrienols in Healthy Fed Humans

**DOI:** 10.4172/2155-9880.1000434

**Published:** 2016-04-28

**Authors:** Asaf A Qureshi, Dilshad A Khan, Neerupma Silswal, Shahid Saleem, Nilofer Qureshi

**Affiliations:** 1 Department of Basic Medical Sciences, University of Missouri-Kansas City, 2411 Holmes Street, Kansas City, MO 64108, USA; 2 Department of Chemical Pathology and Endocrinology, Armed Forces Institute of Pathology, and National University of Medical Science, Rawalpindi, 64000, Pakistan; 3 Pakistan Ordinance Factory Hospital, Wah Cantt, Rawalpindi, 64000, Pakistan; 4 Division of Pharmacology and Toxicology, School of Pharmacy, University of Missouri- Kansas City, 2464 Charlotte Street, Kansas City, MO 64108, USA

**Keywords:** Annatto-based δ-tocotrienol, Bioavailability of AUC, AUMC, MRT, C_max_, T_max_, Vd/l, Cl/h, t_1/2_, Tocopherols, Tocotrienols

## Abstract

**Background:**

Tocotrienols has been known to lower serum lipid parameters below 500 mg/d, while increase lipid parameters at higher dose of 750 mg/d. δ-Tocotrienol has a novel inflammatory property of concentration-dependent inhibition and activation. Therefore, inhibition (anti-inflammatory) property of tocotrienols at low doses is useful for cardiovascular disease, whereas, activation (pro-inflammatory) property using high dose is found effective for treatments of various types of cancer. We have recently described plasma bioavailability of 125 mg/d, 250 mg/d and 500 mg/d doses of δ-tocotrienol in healthy fed subjects, which showed dose-dependent increases in area under the curve (AUC) and maximum concentration (C_max_). Hence, in the current study, higher doses of tocotrienols have used to analyze its effect on plasma pharmacokinetic parameters.

**Aims:**

To evaluate the safety and bioavailability of higher doses (750 mg and 1000 mg) of annatto-based tocotrienols in healthy fed subjects. All four isomers (α-, β-, γ-, δ-) of tocols (tocotrienols and tocopherols) present in the plasmas of subjects were quantified and analyzed for various pharmacokinetic parameters.

**Study design:**

An open-label, randomized study was performed to analyze pharmacokinetics and bioavailability of δ-tocotrienol in 6 healthy fed subjects. All subjects (3/dose) were randomly assigned to one of each dose of 750 mg or 1000 mg. Blood samples were collected at 0, 1, 2, 4, 6, 8 h intervals and all isomers of α-,β-,γ-,δ-tocotrienols, and tocopherols in plasmas were quantified by HPLC.

**Results:**

Oral administration of 750 and 1000 mg/d of tocotrienols resulted in dose-dependent increases in plasmas (ng/ml) AUCt_0-_t_8_ 6621, 7450; AUCt_0-∞_ 8688, 9633; AUMC t_0-∞_ 52497, 57199; MRT 6.04, 5.93; C_max_ 1444, 1592 (P<0.05), respectively, of δ-tocotrienol isomer. Moreover, both doses also resulted in plasmas T_max_ 3.33–4 h; elimination half-life (t_1/2_ h) 2.74, 2.68; time of clearance (Cl-T, l/h) 0.086, 0.078; volume of distribution (Vd/f, mg/h) 0.34, 0.30; and elimination rate constant (ke; h^-1^) 0.25, 0.17, respectively of δ- tocotrienol isomer. Similar results of these parameters were reported for γ-tocotrienol, β- tocotrienol, α-tocotrienol, δ-tocopherol, γ-tocopherol, and β-tocopherol, except for α- tocopherol.

**Conclusions:**

This study has described pharmacokinetics using higher doses of 750 mg/d and 1000 mg/d of δ-tocotrienol. These results confirmed earlier findings that T_max_ was 3-4 h for all isomers of tocotrienols and tocopherols except for α-tocopherol (6 h). These higher doses of tocotrienols were found safe in humans and may be useful for treatments of various types of cancer, diabetes, and Alzheimer's disease.

## Introduction

We have recently reported the pharmacokinetic and bioavailability of various doses (125 mg, 250 mg, and 500 mg) of δ-tocotrienol in 11 fed healthy participants/group (n=33). This was the first study which demonstrated the effect of δ-tocotrienol on several pharmacokinetic parameters of all eight isomers of tocol family (α-, β-, γ-, δ-tocotrienols and α-, β-, γ-, δ- tocopherols) [1]. Our data showed that in plasmas of healthy subjects, δ-tocotrienol bioavailability resulted in dose-dependent increase in area under the curve (AUC) and maximum concentration (C_max_). The time to achieve maximum peak (T_max_) varied between 3-4 h for isomers of tocotrienols and 3-6 h for isomers of tocopherols at 125 mg, 250 mg, 500 mg doses, indicating the longer time of excretion for tocopherols compared to tocotrienols that showed better bioavailability of tocotrienols than tocopherols [1]. The present study is the extension of this study, using higher doses of 750 mg and 1000 mg of tocotrienols.

Tocotrienols have been shown to modulate biological (anti-inflammatory/pro-inflammatory) activity depending on its concentrations [2]. The dose of δ-tocotrienol lower than 500 mg/d specifically decrease the levels of serum total cholesterol, LDL-cholesterol, and triglycerides in a dose-dependent manner, while higher dose of 750 mg/d increase levels of these lipid parameters compared to 250 mg/d [3]. Moreover, low dose mixture of pure α-, γ-and δ-tocotrienols inhibit hepatic β-hydroxy-β-methylglutaryl-coenzyme A (HMG-CoA) reductase activity, whereas high doses induce the activity of HMG-CoA reductase (a rate- limiting enzyme in the biosynthesis of cholesterol) and consequently raise cholesterol [2]. This novel biological property of tocotrienols, at low doses is useful for the control of cardiovascular disease [3], and higher doses would cause apoptotic cell death in various types of cancer [4]. Pure δ-tocotrienol was found to be safe for human consumption even at doses as high as 3,200 mg/d as reported in a recent Phase I Clinical Trial in patients with pancreatic cancer [5]. Thus, δ-tocotrienol is the first naturally-occurring compound, which has the unique dual biological property of inhibition (anti-inflammatory) and activation (pro-inflammatory) depending on its concentration [2-5].

A number of studies have reported the pharmacokinetics and bioavailability of tocotrienols in humans and different animal models. The bioavailability of 300 mg capsule, a mixture of α-tocotrienol + γ-tocotrienol + δ-tocotrienol was administered to fasted and fed subjects (n=8). The plasma T_max_ was found to be between 3-5 h for both food conditions [6]. In addition, 450 mg dose of a mixture of α-tocotrienol rich fraction of barley oil versus γ- tocotrienol rich fraction of palm oil was administered to 7 subjects and plasma T_max_ for α- tocotrienol, β-tocotrienol, γ-tocotrienol, δ-tocotrienol were 2.1∼2.3 h and α-tocopherol was 4.7∼3.1 h [7]. Similarly, in an another human study, plasma T_max_ after administering 2-capsules (300 mg/capsule), which was a mixture of γ-tocotrienol + δ-tocotrienol versus 4-capsules of palm TRF, were 5.64 ± 1.50 h versus 4.73 ± 0.90 h [8].

The intestinal absorption kinetics and the bioavailability of γ-tocotrienol and α-tocopherol have been compared *in vivo* and *in vitro* in rats after administering in the form of oil solution and aqueous medium respectively [9]. There was significantly higher rate of γ-tocotrienol *in vitro* in the aqueous medium form. The oral bioavailability of α-tocopherol (36%) was significantly higher than γ-tocotrienol (9%). The studies *in situ* also revealed significantly higher intestinal permeability for α-tocopherol compared with γ-tocotrienol in rats [9]. These results indicate that intestinal permeability is the main contributing factor for higher bioavailability of γ-tocopherol, and thus by enhancing the γ-tocotrienol permeability would increase its oral bioavailability. Plasma T_max_ for γ-tocotrienol versus α-tocopherol was 2.4 h and 9.7 h respectively [9]. The treatment of mice *in vivo* with δ-tocotrienol also improved radiation-induced alteration in apoptotic and autophagic pathways [10].

**Figure 1:**
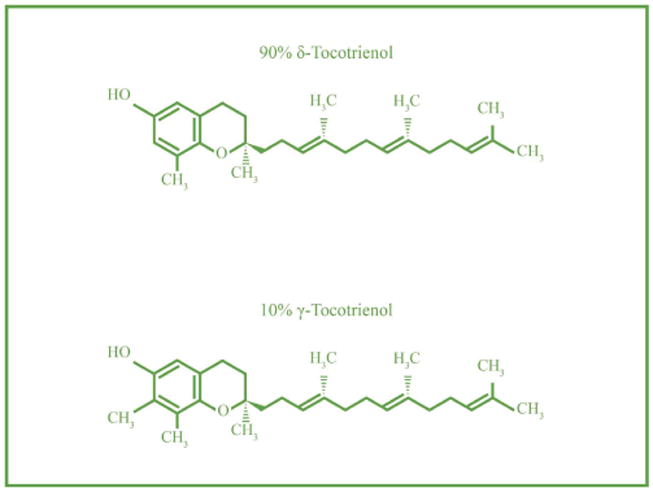
Chemical structures of DeltaGold components (δ-tocotrienol-90% + γ- tocotrienol-10%).

All of these studies using tocotrienols, mixture of tocotrienols, tocotrienol-rich fraction of barley oil (TRF) or tocotrienol-rich fraction of palm oil (TRF, containing α- tocopherol) were carried out using lower doses (300 ∼ 500 mg), except for pancreatic cancer study in humans [5]. Tocotrienols group had plasma T_max_ (2–3 h) and α-tocopherol group had plasma T_max_ (3–5 h). None of the published studies have reported the bioavailability of β-, γ-, δ-tocopherol other than our recently published study describing the pharmacokinetic, bioavailability and absorption of all four isomers of tocotrienols and tocopherols [1]. As reported earlier, the doses below 500 mg/d of α-tocotrienol are effective in lowering lipid parameters by decreasing the level of several lipid parameters while, doses greater than 500 mg/d of δ-tocotrienol (750 mg/d) possibly kill cancer cells [3-5]. Therefore, the present study was carried out using higher doses (750 mg and 1000 mg) of annatto-based tocotrienols (90% δ-tocotrienol + 10% γ-tocotrienol; Figure 1) in human subjects to check the safety and any possible impact of these concentrations. The pharmacokinetic parameters including AUC, C_max_, T_max_ were determined for all isomers (α-, β-, γ-, δ-) of tocotrienols and tocopherols. The results of this study would be able to establish the safe use of higher doses of tocotrienols for the treatment of cancer, diabetes and Alzheimer's disease.

## Materials and Methods

### Reagents

DeltaGold 125 mg soft gels from annatto seeds (composition 90% δ-tocotrienol + 10% γ-tocotrienol) were supplied by American River Nutrition, Inc. (Hadley, MA. USA). The HPLC supplies were purchased as reported recently [1].

### Study subjects

Six healthy subjects (n=3/dose) were selected for the present study. A small number of subjects were used due to results of our earlier study of 11 subjects/dose were very consistent and there was not much variation (<5.0%) between different subjects of the same group [1]. The study was conducted in accordance with the current Good Clinical Practices (FDA, 1996) and the Declaration of Helsinki (WMA, 2008). The study protocol was approved by institutional review board (IRB) of the Pakistan Ordinance Factory (POF) Hospital, Wah Cantt, Pakistan. The study was carried out under FDA approved “Investigating New Drug” (IND) number 36906 as reported earlier [1]. The study was carried out according to the guidelines provided by the United States Food and Drug Administration (FDA, 2003) at Wah Cant, Pakistan. All participants of study signed an informed consent. All subjects were males between the ages of 37-48 years and clinical history and physical examination of all participants was carried out as reported recently [1].

### Study design

An open-label, randomized study was carried out to determine the pharmacokinetics and bioavailability of annatto-based tocotrienol after oral administration in 6 healthy male subjects fed condition. All the subjects were fed Pakistani heavy breakfast comprising of fruit cocktail, Halwa, Puri, Paratha, Omlet, orange juice and tea. The volunteers were randomly assigned to one of DeltaGold soft gels 125 mg/capsule dose levels (750 mg, and 1000 mg), which they received once after a heavy breakfast. The volunteers were instructed not to consume foods rich in vitamin E to minimize variability in intake. Restricted foods included nuts, cereals and vegetable oils. The food was prepared in “Army Mess” under strict hygienic conditions. All subjects were fed very heavy breakfast comprising of Halwa Puri, Paratha, Spanish Omlet, and Lassi (yugart shake), orange juice, or tea; lunch was chicken biryani, Mutton (Lamb) Qorma, Sheermal/Nan, carrot Halva (desert). Dinner was served at the end of study consisting of fried Basmati rice, Kabab (chicken), Rogni Nan, Shahi Tukray, and Kashmiri tea with pistachios. Fruit cocktail was served with every meal as reported recently [1].

### Blood sample collection and extraction

On study day, each subject consumed one dose of δ-tocotrienol with a typical heavy Pakistani breakfast. During the experimental period (0-8 h), subjects consumed good lunch and evening tea as described above. All subjects were allowed to drink water freely. For the determination of the pharmacokinetic profile of tocotrienols in plasma, venous blood samples (2 × 5 ml) were collected in EDTA glazed tubes at pre-dose (0 h) and at post-dose (1 h, 2 h, 4 h, 6 h and 8 h). The samples were then centrifuged at 3000 × g for ten minutes. Processed plasma samples were stored in eppendorf tubes at -80°C till further analysis as reported earlier [1].

### High performance liquid chromatography analysis

The detailed procedure of plasma tocols analyses by high performance liquid chromatography has been described previously [1]. In short, highest purity HPLC grade absolute ethanol and methanol (Fisher Scientific, Pittsburgh, PA). Hexane, Ascorbic Acid and butylated hydroxyl toluene (BHT) were purchased from Sigma Chemical Co. Inc. (St. Louis, MO). The various tocopherols (α-, β-, γ-, δ-) and tocotrienols (α-, β-, γ-, δ-) standards were obtained from ChromaDex Inc. (10005 Muirland Blvd, Suite G, Irvine, CA). The working standards were prepared by mixing appropriate amounts of stock solutions as reported recently [1].

The working standards solutions were prepared of various tocols (1 μg/ml) in hexane; 1% ascorbic in absolute ethanol, 10 mg/10 ml butylated hydroxyl toluene in absolute ethanol. The prepared working stock solutions were kept at -20°C [1]. The extracted plasmas were used for the quantification of α-, β-, γ-, δ-tocotrienols and α-, β-, γ-, δ-tocopherols (tocols) as described earlier [1]. Briefly, plasma (200 μl) was added in screw cap disposable glass tube (15 ml) + 200 μl 1% ascorbic acid + 25 μl butylated hydroxyl toluene (1 mg/1 ml) + 900 μl water + 5 ml hexane, mixed for 10 minutes on a shaker and then centrifuged for 10 min at 5000 rpm. The upper layer was transferred in a centrifuge tube (10 ml) with glass pipette; hexane was removed under vacuum at 40°C using water aspirator. Again hexane (200 μl) was added, vortexed for 30 sec, centrifuged at 2000 rpm for 5 min, and solution was transferred into HPLC injecting vial (0.3 μl). A normal phase silica column (5 micron, 30 cm × 4.0 mm I.D. obtained from Waters Associates, Milford, MA. USA) attached to a Guard column was used to separate various tocols. HPLC system consisted of a continuous-flow 307 pump (Gilson, Madison, Wisconsin, USA) and mobile phase was pumped at a flow rate of 1.3 ml/min depending upon the elution of tocols. α- Tocopherol was eluted within 5-6 min and δ-tocotrienol between 18-20 min, pressure varied between 0.4-0.5 psi under these conditions. Fluorescence was read with Shimadzu fluorescence monitor (Model RF-535) set at excitation wavelength of 296 nm and an emission wavelength of 330 nm.

The peak areas were determined by Shimadzu Integrator- Model C-R3A (Shimadzu, Wood Dale, IL. USA). The eluting solvent was 0.3% (vol/vol) isopropyl alcohol in hexane. The extracted sample (20 μl) was introduced into the column through the 10 μl loop of Gilson's autosampler-231 injector. All the samples were analyzed in duplicate or triplicate. The retention time of the individual peaks of the unknown tocols were compared against the retention time of the pure standard tocols. The tocols were eluted under these conditions in the sequence: α-tocopherol, α-tocotrienol, β-tocopherol, β-tocotrienol, γ-tocopherol, γ-tocotrienol, δ-tocopherol, δ-tocotrienol [1].

### Pharmacokinetic analysis

The computer software PK Solver 2.0 was used for calculation of pharmacokinetic parameters through standard two compartmental analysis of each subject in each group (for AUCt_0_ – t_8_, AUCt_0-∞_, AUMCt_0-∞_, MRT, C_max_, T_max_, half-life time (t_1/2_), time of clearance (Cl-T). The values for these parameters were based on mean concentrations of each time point of each isomer (α-, β-, γ-, δ-) of tocotrienols (T3) and tocopherols (T) and also using the values of each subject in each group. Data of plasma tocotrienols concentration versus time was used to calculate pharmacokinetic parameters. Area under the curve (AUC) from 0 h- time to 8 h-time (AUCt_0_ – t_8_ h) for plasma tocotrienols was calculated by trapezoidal rule, where “t” is the last measured time point. The maximum serum concentration (C_max_) and the time to reach peak (T_max_) were obtained directly by inspecting each individual plasma level- time curves. The Ke values were calculated by equation: time of clearance (Cl-T)/Vd/f) [1].

### Statistical analysis

Descriptive statistics, comprising mean and SD were calculated by using GraphPad Prism 5.0. Percent differences were calculated from baseline value of each analyte. Repeated one-way ANOVA was applied on dose response data. P value of ≤0.05 was considered significant. Data are reported as mean ± SD (Standard Deviation).

## Results

This is the first report describing the quantitative determination of all eight isomers of tocol (four tocotrienols plus four tocopherols) from the human plasma samples obtained after administering higher doses (750 mg and 1000 mg) of annatto-based δ-tocotrienol. The physical characteristics of all participants are reported in Table 1. The ages, heights, weights, systolic, diastolic blood pressures, and other parameters of participants (fed each dose) were closely similar to avoid variation in the results (Table 1).

**Figure 2:**
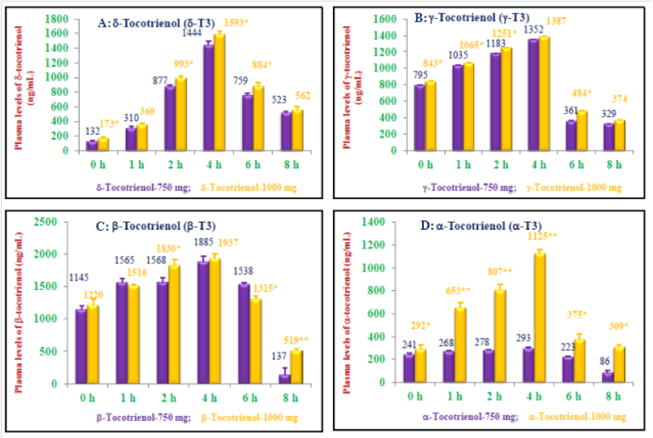
Plasma peak concentrations (C_max_, ng/ml) of δ-, γ-, β, α-tocotrienols of various doses: The single dose of 750 mg or 1000 mg of DeltaGold-based δ- tocotrienol was administered in one day to healthy fed subjects. The plasma samples were collected at different time points from each subject, and processed to carry out normal phase HPLC analyses as described in Materials and Methods section. 2A: The plasma concentration of δ-tocotrienol at 0 h, 1 h, 2 h, 4 h, 6 h, and 8 h time points. 2B: The plasma concentration of γ-tocotrienol at 0 h – 8 h time points. 2C: The plasma concentration of β-tocotrienol at 0–8 h time points. 2D: The plasma concentration of α-tocotrienol at 0–8 h time points. Values are means ± standard deviation (n=3/dose). Values are significantly diferent compared to 750 mg dose versus 1000 mg dose at *P<0.05; or **P<0.01 from each other.

HPLC was carried out as reported earlier by using normal phase silica of plasma samples collected at 0 h, 1 h, 2 h, 4 h, 6 h, and 8 h (7 samples) of each subject for the determination of pharmacokinetics of various tocols [1]. HPLC analyses of plasma samples of doses of 750 mg and 1000 mg showed the elution of all isomers of tocols (α-, β-, γ-, δ-tocotrienols and α-, β-, γ-, δ-tocopherols) for each time intervals (0–8 h). The tocotrienols (ng/ml) present in 750 mg dose were β-tocotrienol (7838)>γ-tocotrienol (5055)>δ- tocotrienol (4045)>α-tocotrienol (1389), whereas for tocopherols was δ-tocopherol (13117)>γ-tocopherol (5544)>β-tocopherol (3269)>γ-tocopherol (2188) as shown in Table 2 and Figure 2.

**Figure 3:**
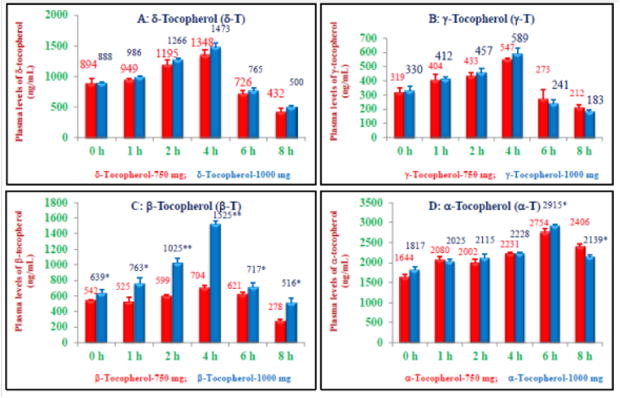
Plasma peak concentrations (C_max_, ng/ml) of δ-, γ-, β-, α-tocopherols of various doses: The doses of 750 mg or 1000 mg/d of DeltaGold-based δ-tocotrienol were administered to healthy fed subjects. The plasma samples were collected at different time points from each subject, and processed to carry out normal phase HPLC analyses as described in Materials and Methods section. 3A: The plasma concentration of δ- tocopherol at 0 h, 1 h, 2 h, 4 h, 6 h, and 8 h time points. 3B: The plasma concentration of γ-tocopherol at 0–8 h time points. 3C: The plasma concentration of β-tocopherol at 0–8 h time points. 3D: The plasma concentration of α-tocopherol at 0–8 h time points. Values are means ± standard deviation (n=3/dose). Values are significantly different compared to 750 mg dose versus 1000 mg dose at *P<0.05; or **P<0.01 from each other.

Similar pattern of concentrations (ng/ml) of tocols was observed in plasmas obtained from subjects that were fed 1000 mg dose, and tocotrienols (ng/ml) were β-tocotrienol (8337)>γ-tocotrienol (5404)>δ-tocotrienol (4465)>α-tocotrienol (3562), and tocopherols concentrations order was α-tocopherol (13239)>δ-tocopherol (5838)>β-tocopherol (5185)>γ-tocopherol (2212) (Table 3 and Figure 3). None of the subjects reported any adverse effects at the end of the study, which shows that tocotrienols were deemed safe even at 1000 mg dose.

The analyses of important pharmacokinetic parameters for all different isomers were calculated for each time interval/subject/group by PK Solver 2.0, which provided values with standard deviations (SD) for each group. The pharmacokinetics and bioavailability of a compound required analysis of plasma for determination of area under the concentration-time curve (AUCt_0_ – t_8_ or ∞), AUMCt_0-∞_, Mean Residence Time (MRT), plasma maximum concentration (C_max_), time to reach plasma concentration (T_max_), the apparent volume of distribution (Vd/l), time of clearance (Cl-T, Cl/h), half-life time (t_1/2_), and elimination rate constant (ke).

The δ-tocotrienol feeding showed dose-dependent increases in plasmas of subjects area under the curve, AUCt_0_ - t_8_ (ng/ml) for 750 mg (6621 ± 50) and 1000 mg (7450 ± 89) which statistically significant (P<0.05) (Tables 4 and 5). Similar increases in AUC_0-∞_ (ng/ml), 8688 ± 201, 9633 ± 383 (P<0.05); AUMCt_0-∞_ (ng/ml), 52497 ± 2096, 57199 ± 5007, and MRT (h) 6.04 ± 0.14, 5.93 ± 0.34, were observed for doses of 750 mg and 1000 mg, respectively (Tables 4 and 5). Plasmas peak concentration (C_max_, ng/ml), 1444 ± 53, 1592 ± 44; time to achieve plasma peak (T_max_), 3.33, 4.0 h; elimination half-life (t_1/2_ h), 2.74 ± 0.13, 2.68 ± 0.29 h; time of clearance (Cl-T, h), 0.086 ± 0.002, 0.078 ± 0.003; apparent volume of distribution (Vd, ml), 0.341 ± 0.012, 0.300 ± 0.021; and elimination rate constant (ke; h^-l^), 0.253 ± 0.167, 0.260 ± 0.143 for doses of 750 mg, and 1000 mg, respectively, were calculated as shown in Tables 4A and 5A Most of them were significantly different (P<0.05).

Similar increases in all the parameters were observed in plasmas for γ-tocotrienol, such as AUCt_0_ – t_8_ (ng/ml) 6962 ± 98, 7480 ± 129, (P<0.05), for β-tocotrienol, 11474 ± 316, 11895 ± 231, and for α-tocotrienol, 198 ± 102, 548 ± 19, P<0.05 for doses of 750 mg, and 1000 mg, respectively as shown in Tables 4A and 5A. The plasma peak concentrations (C_max_ ng/ml) for γ-tocotrienol, 1352 ± 28, 1387 ± 13; β-tocotrienol, 1885 ± 91, 1948 ± 91; and α-tocotrienol, 30 ± 1, 116 ± 4 (P<0.05) were observed for 750 mg, and 1000 mg, respectively (Table 4A and 5A). The time to achieve plasma maximum peaks (T_max_ h) were for γ-tocotrienol, 750 mg (4 h), 1000 mg (4 h); for β-tocotrienol were 750 mg (4 h), 1000 mg (3.33 h), and similarly, for α-tocotrienol were 750 mg (3.33 h), and 1000 mg (4 h) for these isomers, as shown in Tables 4A and 5A.

The highest AUCt_0_ – t_8_ (ng/ml) 11474, 11892 and C_max_ (ng/ml) 1885, 1948 values for both doses were found with β-tocotrienol and lowest values were with α-tocotrienol, AUCt_0_ – t_8_ (ng/ml) 198, 548 and C_max_ (ng/ml) 1885, 1948 were with 750 mg and 1000 mg doses, respectively. The maximum peak elution time was between 3 h to 4 h for both doses (Tables 4A and 5A).

The values for all these parameters showed dose-dependent decreases and most of them were significantly different at P<0.05–0.01 as reported in Tables 4A and 5A. There was also dose-dependent increases of pharmacokinetic parameters of δ-tocopherol; AUCt_0_ – t_8_ (7767 ± 192, 8306 ± 217, ng/ml, P<0.05); AUCt_0_ – t∞ (9295 ± 232, 10169 ± 69, ng/ml, P<0.01); AUMC t_0–∞_ (45137 + 3349, 51640 + 2782, ng/ml, P<0.05); MRT (4.85 + 0.27, 6.04 + 0.14 h, P<0.05); C_max_, (1354 ± 80, 1475 ± 71) as shown in Tables 4B and 5B, and T_max_ were 3.33, 4.0 h, for doses 750 mg and 1000 mg, respectively.

Similarly, for γ-tocopherol, AUCt_0_ – t_8_ h (3066 ± 188, 3108 ± 148 ng/ml); AUCt_0-∞_ (3960 ± 252, 3739 ± 192 ng/mL); AUMC t_0-∞_ (22002 ± 2113, 18102 ± 1302, ng/ml); MRT (5.55 ± 0.24, 4.84 ± 0.15 h,); C_max_ (548 ± 12, 589 ± 39 ng/ml) showed dose-dependent increases that were in-significant, while T_max_ was 4 h for both doses (Tables 4B and 5B). For β-tocopherol, AUCt_0_ – t_8_ h (4623 ± 82, 7221 ± 184 ng/ml); AUCt_0-∞_ (4624 ± 82, 7221 ± 184 ng/ml; AUMC t_0-∞_ (32589 ± 2556, 54126 ± 5921, ng/ml, P<0.01) showed significant dose-dependent increases, whereas, values for MRT (5.58 ± 0.31, 5.74 ± 0.31 h), C_max_ (704 ± 28, 1325 ± 56 ng/ml, P<0.01) showed dose dependent increases for 750 mg and 1000 mg and T_max_ was 4 h for both doses (Tables 4B and 5B).

**Figure 4:**
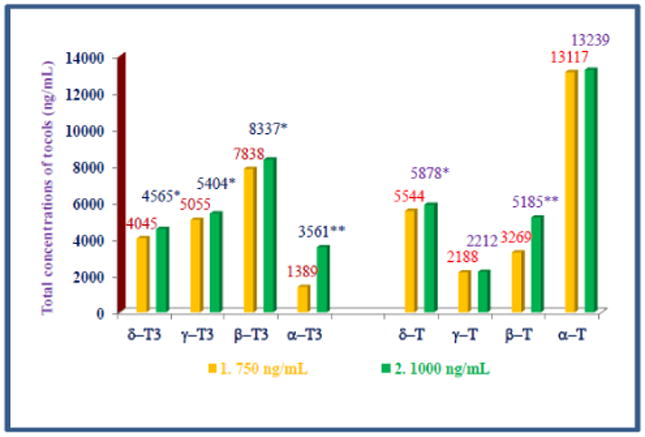
Total plasma concentrations (ng/ml) of four isomers of tocotrienols and tocopherols of various doses: The doses of 750 mg or 1000 mg/d of DeltaGold-based δ- tocotrienol were administered to healthy fed subjects. The plasma samples were collected at different time points and processed to carry out normal phase HPLC analyses as described in Material and Method section. The total plasma concentrations of each isomer of tocotrienols and tocopherols were compared for each dose. Values are means ± standard deviation (n=3/dose). Values are significantly different compared to 750 mg dose versus 1000 mg dose at *P<0.05; or **P<0.01 from each other.

**Figure 5:**
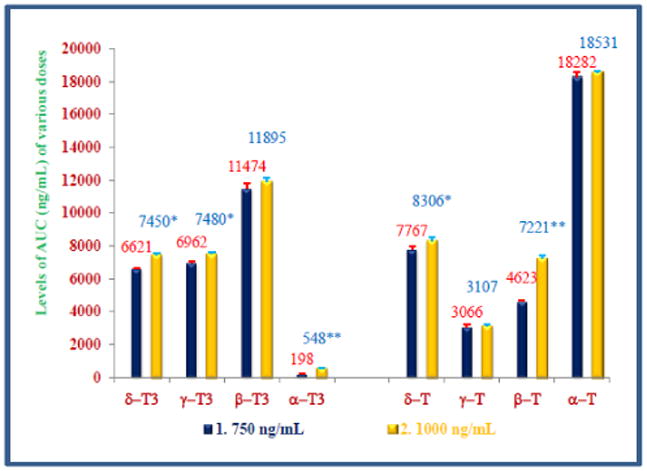
Plasma levels of AUC (ng/ml) of tocols of various doses: The doses of 750 mg and 1000 mg/d of DeltaGold-based δ-tocotrienol were administered to healthy fed subjects. The plasma samples were collected at different time points and processed to carry out normal phase HPLC analyses as described in Material and Method section. The plasma levels of AUC of each isomer of tocotrienols and tocopherols were analyzed by using PK Solver 2.0, and then compared for each dose. Values are means ± standard deviation (n=3/dose). Values are significantly different compared to 750 mg dose versus 1000 mg dose at *P<0.05; or **P<0.01 from each other.

The maximum values for AUCt_0_ – t_8_ h (18282 ± 275, 18531 ± 97 ng/ ml); C_max_ (2754 ± 84, 2915 ± 39 ng/ml, P<0.05) were observed with α-tocopherol and T_max_ was 6 h for both doses (Tables 4B and 5B). The summary of pharmacokinetic parameters of all four tocotrienols and tocopherols are summarized in Tables 4A, 4B, 5A and 5B. The total concentrations of each isomer of tocotrienols (α-, β-, γ-, δ-) and tocopherols (α-, β-, γ-, δ-) for each dose were compared in Figure 4, which showed the highest plasma concentrations for α-tocopherol. Similarly, AUCt_0_ – t_8_ h (ng/ml) for α-tocopherol was greatest out of all four tocotrienols and tocopherols when compared for 750 mg and 1000 mg doses as shown in Figure 5.

## Discussion

The present study describes effects of higher doses of annatto-based δ-tocotrienol (without α-tocopherol) on the pharmacokinetic parameters of all eight isomers of tocol family (α-, β-, γ-, δ-tocotrienols and α-, β-, γ-, δ-tocopherols) in healthy fed subjects. The doses 750 mg and 1000 mg of δ-tocotrienol fed resulted in dose-dependent increased levels of all eight isomers of tocols in the plasma and their values of AUCt_0_ – t_8_, C_max_, and T_max_. In addition to these three parameters, there were dose dependent increases in AUCt_0-∞_, AUMC t_0-∞_, MRT, half-life time (t_1/2_), time of clearance (Cl-T), the apparent volume of distribution (Vd/f), and elimination rate constant (Ke) for all isomers of tocols except for α-tocopherol. T_max_ for all the isomers of tocols was 4 h except for α-tocopherol (6 h). These results are in line with our earlier findings using lower doses of tocotrienols [1]. These pharmacokinetic parameters were not calculated for α-tocopherol due to lack of values for one more time point after plasma peak was achieved. It would have been better had we collected blood samples for one more time point at (10 h). Pharmacokinetic results from our previous study using low doses of δ-tocotrienol showed standard deviations less than 5% in tocols therefore in the current study we have used 3 subjects /dose and still the standard deviation was less than 5% except AUMC t_0-∞_ [1]. Most of the published pharmacokinetics studies have been carried out by using 3-8 subjects/dose [5-9].

The main difference between our earlier results of lower doses with feeding125 mg, 250 mg, and 500 mg versus present results of higher doses were that there were no peaks for δ-tocotrienol and δ-tocopherol at time point 0 h and 1 h in plasma samples with 125 mg dose. While plasma samples of 250 mg and 500 mg doses there were also any peaks at 0 h time point of δ-tocotrienol and δ-tocopherol [1]. The peaks of α-tocopherol, β-tocopherol, γ- tocopherol, and δ-tocotrienol were observed at 0 h time point for the samples of all (125 mg, 250 mg, and 500 mg) doses [1]. In contrast, plasma samples obtained with feeding subjects with higher doses (750 mg and 1000 mg) resulted in elution of all isomers (α-, β-, γ-, δ- tocotrienols and α-, β-, γ-, δ-tocopherols) for each time point between 0 h – 8 h. Overall, the consumption of the higher doses (750 mg and 1000 mg) of annatto-based tocotrienols are safe for normal healthy humans and tocotrienols at high doses between 200 mg and 3200 mg daily are also well tolerated without toxicity in pancreatic cancer patients [5].

Tocotrienols are lipophilic in nature, therefore poorly soluble and show limited absorption and low bioavailability as reported earlier [6,9]. Therefore, several methods have been used to improve the oral intestinal bioavailability of many lipophilic compounds using lipid-based formulations [11-13]. It was reported that co-administration of tocotrienols with lipids causes a delay in the rate of gastric emptying which enhances drug solubility by stimulating the secretion of bile salts and phospholipids into the gastrointestinal tract, which therefore increase absorption and bioavailability of tocotrienols [14,15]. These lipid-based formulations also increase the absorption of lipophilic compounds into lipoproteins, which allow more lymphatic absorption [16-18] and thereby bypassing hepatic portal system metabolism [19-21].

Although, it is difficult to determine the absolute bioavailability of tocotrienols in humans, but relative bioavailability of γ-tocotrienols increases 3.5-fold when administered with food which has fat as a major component [6]. While, in rats, δ-tocotrienol and γ-tocotrienol, oral bioavailability was found to be as low as 8.5% and 9% respectively [9]. In order to achieve increase bioavailability, a self-emulsifying drug delivery systems (SEDDS) have been used to promote the oral absorption of poorly water-soluble drugs [11]. A recent study reported the bioavailability of γ-tocotrienol, δ-tocotrienol versus annatto-based γ-tocotrienol (10%) + δ-tocotrienol (90%) dissolved in SEDDS using rats and displayed that γ-tocotrienol (10%) and δ-tocotrienol (90%) showed bioavailability of 332 (0.05 mg/kg) and 31.5 (0.5 mg/kg) for low doses, and bioavailability was 4.5 ± 0.1 (2.5 mg/kg) and 3.0 ± 0.1 (25 mg/kg) at higher doses, due to nonlinear absorption kinetics [11]. The results of γ- tocotrienol dissolved in SEDDS versus commercial Tocovid were also reported *in vitro* and *in vivo* in rats [22]. The *in vitro* results showed two-fold increase in the solubilization as well as higher cellular uptake while *in vivo* studies showed two-fold improvement in oral bioavailabity of γ-tocotrienol in SEDDS formulations compared to Tocovid [22].

In the current study, we have recruited only males to avoid any genetic variability. There are several factors like age, drug-drug interaction, disease, environmental pollutants, and genetics which can contribute to variability in our pharmacokinetics results. In this study, we tried to take care of those factors by selecting the subjects in the same age group, with no past history of major disease, as well as taking no drugs. As food an example of environmental factor contributing to variability in our results, the subjects have to eat large quantities of heavy breakfast or lunch, during eight hour period to have any dietary effect on the total concentrations of tocotrienols and tocopherols.

## Conclusions

This study describes the effects of higher doses of 750 mg and 1000 mg of annatto-based tocotrienols on the plasma pharmacokinetic parameters of all eight isomers of tocols in humans. The bioavailability of α-, β-, γ-, δ-tocotrienols and α-, β-, γ-, δ-tocopherols resulted dose-dependent increases in plasma AUCt_0_-_8_, AUCt_0-∞_, AUMCt_0_-_∞_, C_max_. The T_max_ was 3-4 h for all isomers, except α-tocopherol (6 h) for doses of 750 mg and 1000 mg.

The study participants did not report any adverse effects with higher dose feeding at the end of the study and were safely tolerated by them. Moreover, the novel property of tocotrienols, concentration-dependent inhibition and activation, observed in case of cardiovascular parameters (low doses, below 500 mg), and cancer patients (higher doses, above 500 mg) had no impact on T_max_ of these higher doses (750 mg and 1000 mg) in humans. Moreover, it is better to take tocotrienols after lunch/dinner (food) to increase their permeability, absorption, and bioavailability [6]. These high doses (750 mg and 1000 mg) are safe of tocotrienols and may be useful for the treatment of various types of cancer, diabetes, and Alzheimer's disease in humans.

## Figures and Tables

**Table 1:** Baseline characteristics of pharmacokinetic study subjects (n=3/treatment).

#	Parameters	δ -Tocotrienol (750 mg)	δ -Tocotrienol (1000 mg)
1	Age (Years)	42.33 ± 5.51*	34.42 ± 2.43*
2	Males (n)	3	3
3	Height (cm)	171.22 ± 3.16	173.25 ± 3.43
4	Weight (kg)	77.33 ± 4.16	78.04 ± 1.55
5	BMI (kg/m^2^)	28.73 ± 1.43	27.97 ± 1.46
6	Pulse (Min)	78.33 ± 2.52	75.62 ± 2.63
7	Systolic BP (mmHg)	127.51 ± 1.17	124.79 ± 1.87
7	Diastolic BP (mmHg)	83.95 ± 1.03	83.29 ± 1.88
8	Serum Creatinine (mmol/l)	97.58 ± 2.40	95.04 ± 1.67
9	Serum ALT (U/l)	21.92 ± 1.59	28.20 ± 1.63
10	Serum Glucose (mmol/l)	4.44 ± 0.32	4.19 ± 0.28
11	Serum Total Cholesterol (mmol/l)	4.75 ± 0.90	5.18 ± 0.18
12	Serum Triglycerides (mmo/l)	1.47 ± 0.16	1.49 ± 0.07

* Values represent ± Standard deviation (SD)

**Table 2:** Estimation of plasma tocols by normal phase HPLC of pharmacokinetic human study after feeding single dose of 750 mg of δ -tocotrienol in one day.

Subjects: 1-3.	Normal Phase-Silica column.							
Tocols-----→	δ-Tocotrienol	γ Tocotrienol	β-Tocotrienol	α -Tocotrienol	δ-Tocopherol	γ -Tocopherol	β -Tocopherol	α -Tocotrienol
Hour	ng/ml	ng/ml	ng/ml	ng/ml	ng/ml	ng/ml	ng/ml	ng/ml
0 hr.	132 ± 12*	795 ± 19*	1145 ± 61*	241 ± 16*	894 ± 69*	319 ± 30*	542 ± 11*	1644 ± 59*
1 hr.	310 ± 28	1035 ± 10	1565 ± 62	268 ± 13	949 ± 11	404 ± 44	525 ± 54	2080 ± 68
2 hr.	877 ± 24	1183 ± 16	1568 ± 77	278 ± 7	1195 ± 69	433 ± 26	599 ± 14	2002 ± 91
4 hr.	1444 ± 53	1352 ± 23	1885 ± 91	293 ± 11	1348 ± 93	547 ± 12	704 ± 29	2231 ± 35
6 hr.	759 ± 30	361 ± 74	1538 ± 22	223 ± 5	726 ± 51	273 ± 67	621 ± 28	2754 ± 84
8 hr.	523 ± 13	329 ± 17	137 ± 102	86 ± 16	432 ± 45	212 ± 16	278 ± 16	2406 ± 51
Total tocols (ng/ml)	4045	5055	7838	1389	5544	2188	3269	13117

* Values represent ± Standard Deviation (± SD).

**Table 3:** Estimation of tocols by normal phase HPLC of pharmacokinetic human study after feeding single dose of 1000 mg of δ -tocotrienol in one day.

Subjects: 4-6.	Normal Phase-Silica column (NP).							
Tocols-----→	δ -Tocotrienol	γ -Tocotrienol	β -Tocotrienol	α -Tocotrienol	δ -Tocopherol	γ -Tocopherol	β -Tocopherol	α -Tocopherol
Hour	ng/ml	ng/ml	ng/ml	ng/ml	ng/ml	ng/ml	ng/ml	ng/ml
0 hr.	173 ± 11*	843 ± 13*	1220 ± 93*	292 ± 39*	888 ± 22*	330 ± 34*	639 ± 38*	1817 ± 80*
1 hr.	360 ± 20	1065 ± 7	1516 ± 21	653 ± 42	986 ± 18	412 ± 17	763 ± 69	2025 ± 66
2 hr.	993 ± 33	1251 ± 32	1830 ± 91	807 ± 49	1266 ± 25	457 ± 33	1025 ± 58	2115 ± 92
4 hr.	1593 ± 44	1387 ± 10	1937 ± 72	1125 ± 35	1473 ± 71	589 ± 39	1525 ± 32	2228 ± 22
6 hr.	884 ± 45	484 ± 17	1315 ± 48	375 ± 49	765 ± 44	241 ± 28	717 ± 56	2915 ± 35
8 hr.	562 ± 47	374 ± 27	519 ± 20	309 ± 17	500 ± 25	183 ± 14	516 ± 51	2139 ± 61
Total tocols (ng/ml)	4565	5404	8337	3561	5878	2212	5185	13239

* Values represent ± Standard Deviation (± SD).

**Table 4:** Pharmacokinetic parameters after feeding single dose of 750 mg of δ-tocotrienol in one day.

A:	δ-Tocotrienol	γ- Tocotrienol	β -Tocotrienol	α-Tocotrienol
1. Area Under the Curve-t_0_-t_8_ (AUCt_0-8_; ng/ml)	6620.87 ± 49.67^a^*	6961.92 ± 97.55^b^	11473.96 ± 316.15^c^	197.89 ± 1.02^d^*
2. Area Under the Curve-t_0_- (AUCt_0_-_∞_; ng/ml)	8687.69 ± 201.01^a^ *	7895.14 ± 73.43^b^	11709.23 ± 459.66^c^	225.50 ± 6.79^d^*
3. Cumulative Area Under the Curve-t_0_-_∞_ (AUMCt_0_-_∞_; ng/ml)	52496.47 ± 2095.81^a^	32479.70 ± 606.11^b^	43200.35 ± 3122.43^c^	1009.47 ± 88.31^d^
4. Mean Residence Time (/h)	6.04 ± 0.139^a^	4.11 ± 0.049^b^	3.69 ± 0.16^b^	4.47 ± 0.26^b^
5. Peak Plasma Concentration (C_max_; ng/ml)	1444.23 ± 53.07^a^	1352.41 ± 28.14^b^	1885.20 ± 90.95^c^	30.25 ± 1.06^d^
6. Time to achieve plasma peak (T_max_; h)	4.00^a^	4.00^b^	4.00^c^	3.33 + 1.16^a^
7. Elimination of Half-life time (t1/2; h)	2.74 ± 0.13^a^	1.96 ± 0.06^b^	1.02 ± 0.34^c^	2.21 ± 0.18^a^
8. Time of clearance (Cl-T; I/h)	0.086 ± 0.002^a^	0.095 ± 0.001^a^	0.064 ± 0.003^b^	3.33 ± 0.102^c^
9. Apparent volume of distribution (Vd/f; ml)	0.341 ± 0.012^a^	0.269 ± 0.008^b^	0.094 ± 0.029^c^	10.583 ± 0.543^d^
10. Elimination rate constant (ke; h^-1^)	0.253 ± 0.167^a^	0.353 ± 0.125^a^	0.681 ± 0.103^b^	0.315 ± 0.188^a^
B:	δ -Tocopherol	γ-Tocopherol	β -Tocopherol	α-Tocopherol
1. Area Under the Curve-t_0_-t_8_ (AUCt_0_-8; ng/ml)	7766.79 ± 192.42^a^*	3066.14 ± 187.49^b^	4623.38 ± 81.72^c^	18282.03 ± 275.20^d^*
2. Area Under the Curve-t_0-∞_ (AUCt_0 -∞_; ng/ml)	9294.91 ± 232.20^a^*	3960.39 ± 251.48^b^	5831.54 ± 141.01^c^	
3. Cumulative Area Under the Curve-t_0_-_∞_ (AUMCt_0_-_∞_)	45136.59 ± 3348.58^a^	22002.09 ± 2112.78^b^	32588.53 ± 2555.89^c^	
4. Mean Residence Time (/h)	4.85 ± 0.269^a^	5.55 ± 0.244^b^	5.58 ± 0.314^b^	
5. Peak Plasma Concentration (C_max_; ng/ml)	1353.79 ± 79.45^a^	547.45 ± 11.77^b^	704.16 ± 28.18^c^	2754.36 ± 83.72^d^
6. Time to achieve plasma peak (T_max_; h)	3.33 ± 1.16^a^	4.00^b^	4.00^a^	6.00^b^
7. Elimination of Half-life time (t_1/2_; h)	2.44 ± 0.19^a^	2.92 ± 0.21^b^	3.02 ± 0.32^b^	
8. Time of clearance (Cl-T; I/h)	0.081 ± 0.002^a^	0.190 ± 0.012^b^	0.129 ± 0.003^c^	
9. Apparent volume of distribution (Vd/f; ml)	0.284 ± 0.021^a^	0.799 ± 0.047^b^	0.556 ± 0.046^c^	
10. Elimination rate constant (ke; h^-1^)	0.286 ± 0.095^a^	0.238 ± 0.255^b^	0.232 ± 0.065^b^	

a-d Values in a row not sharing a common letter are significantly different at P<0.05-0.01.

* Values represent ± Standard Deviation (SD).

**Table 5:** Pharmacokinetic parameters after feeding single dose of 1000 mg of δ -tocotrienol in one day.

A:	δ -Tocotrienol	γ -Tocotrienol	β -Tocotrienol	α -Tocotrienol	
1. Area Under the Curve-t_0_-t_8_ (AUCt_0-8_; ng/ml)	7450.10 ± 89.01^a^*	7479.89 ±129.37^a^	11895.22 ± 231.01^b^	547.58 ±19.06^c^*	
2. Area Under the Curve-t_0-∞_ (AUCt_0-∞_; ng/ml)	9633.18 ± 382.98^a^*	8626.41 ± 277.17^b^	13475.36 ± 258,61^c^	646.41 ± 25.09^d^*	
3. Cumulative Area Under the Curve-t_0-∞_-(AUMCt_0-∞_; ng/ml)	57198.99 ± 5006.46^a^	37413.68 ± 2525.63^a^	59888.88 ± 1767.19^a^	3059/45 ± 178.82^c^	
4. Mean Residence Time (/h)	5.93 ± 0.364^a^	4.33 ± 0.159^b^	4.44 ± 0.076^b^	4.73 ± 0.123^b^	
5. Peak Plasma Concentration (C_max_; ng/ml)	1591.89 ± 43.97^a^	1386.99.41 ± 12.49^b^	1948.13 ± 66.43^c^	115.84 ± 3.57^d^	
6. Time to achieve plasma peak (T_max_; h)	4.00^a^	4.00^a^	3.33 ± 1.16^a^	4.00^a^	
7. Elimination of Half-life time (t_1/2_; h)	2.68 ± 0.29^a^	2.12 ± 0.14^b^	2.11 ± 0.03^b^	2.15 ± 0.13^b^	
8. Time of clearance (Cl-T; I/h)	0.078 ± 0.003^a^	0.116 ± 0.004^b^	0.074 ± 0.001^a^	3.33 ± 0.102^c^	
9. Apparent volume of distribution (Vd/f; ml)	0.300 ± 0.021^a^	0.354 ± 0.014^b^	0.226 ± 0.006^c^	4.797 ± 0.232^d^	
10. Elimination rate constant (ke; h^-1^)	0.260 ± 0.143^a^	0.328 ± 0.286^a^	0.327 ± 0.167^a^	0.694 ± 0.443^a^	
B:	δ -Tocopherol	γ -Tocopherol	β -Tocopherol	α -Tocopherol	
1. Area Under the Curve-t_0_-t_8_ (AUCt_0-8_; ng/ml)	8305.81 ± 216.66^a^*	3107.38 ± 147.51^b^	7220.65 ± 183.49^c^	18531.38 ± 96.78^d^*	
2. Area Under the Curve-t_0-∞_ (AUCt_0-∞_; ng/ml)	10169.12 ± 68.88^a^*	3739.17 ± 191.92^b^	9417.46 ± 531.19^b^		
3. Cumulative Area Under the Curve-t_0-∞_ (AUMCt_0-∞_; ng/ml)	51639.53 ± 2781.56^a^	18102.24 ± 1302.29^b^	54126.15 ± 5920.86^a^		
4. Mean Residence Time (/h)	5.08 ± 0.251^a^	4.84 ± 0.145^a^	5.74 ± 0.310^b^		
5. Peak Plasma Concentration (C_max_; ng/ml)	1472.84 ± 71.21^a^	589.38 ± 39.07^b^	1325.03 ± 55.60^a^	2914.95 ± 39.04^c^	
6. Time to achieve plasma peak (T_max_; h)	4.00^a^	4.00^a^	4.00^a^	6.00^b^	
7. Elimination of Half-life time (t_1/2_; h)	2.58 ± 0.22^ab^	2.38 ± 0.19^a^	2.94 ± 0.19^b^		
8. Time of clearance (Cl-T; I/h)	0.098 ± 0.001^a^	0.190 ± 0.012^a^	0.129 ± 0.003^b^		
9. Apparent volume of distribution (Vd/f; ml)	0.366 ± 0.030^a^	0.920 ± 0.070^b^	0.450 ± 0.004^c^		
10. Elimination rate constant (ke; h^-1^)	0.268 ± 0.033^a^	0.207 ± 0.171^b^	0.287 ± 0.750^c^		

a-d Values in a row not sharing a common letter are significantly different at P<0.01-0.001.

* Values represent ± Standard Deviation (SD).

## Abbreviations

AUC: Area Under the Concentration Curve
AUMC: Cumulative Area under the Curve
MRT: Mean Residence Time
C_max_: Peak Concentration Maximum
T_max_: Time to Reach Maximum Concentration
Vd/l: Apparent Volume of Distribution
Cl-T (Cl/h): Time of Clearance
t_1/2_ (h): Half-life Time
ke (h^-1^): Elimination Rate Constant
